# Sonic Hedgehog Intron Variant Associated With an Unusual Pediatric Cortical Cataract

**DOI:** 10.1167/iovs.63.6.25

**Published:** 2022-06-24

**Authors:** Terri L. Young, Kristina N. Whisenhunt, Sarah M. LaMartina, Alex W. Hewitt, David A. Mackey, Stuart W. Tompson

**Affiliations:** 1 Department of Ophthalmology and Visual Sciences, University of Wisconsin-Madison, Madison, Wisconsin, United States; 2 Centre for Eye Research Australia, Royal Victorian Eye and Ear Hospital, East Melbourne, Victoria, Australia; 3 Lions Eye Institute, Centre for Ophthalmology and Visual Science, University of Western Australia, Perth, Western Australia, Australia; 4 Eye Department, Royal Hobart Hospital, University of Tasmania, Hobart, Tasmania, Australia

**Keywords:** sonic hedgehog, intron, enhancer, pediatric, cataract

## Abstract

**Purpose:**

To identify the genetic basis of an unusual pediatric cortical cataract demonstrating autosomal dominant inheritance in a large European–Australian pedigree.

**Methods:**

DNA from four affected individuals were exome sequenced utilizing a NimbleGen SeqCap EZ Exome V3 kit and HiSeq 2500. DNA from 12 affected and four unaffected individuals were genotyped using Human OmniExpress-24 BeadChips. Multipoint linkage and haplotyping were performed (Superlink-Online SNP). DNA from one affected individual and his unaffected father were whole-genome sequenced on a HiSeq X Ten system. Rare small insertions/deletions and single-nucleotide variants (SNVs) were identified in the disease-linked region (Golden Helix SVS). Combined Annotation Dependent Depletion (CADD) analysis predicted variant deleteriousness. Putative enhancer function and variant effects were determined using the Dual-Glo Luciferase Assay system.

**Results:**

Linkage mapping identified a 6.23-centimorgan support interval at chromosome 7q36. A co-segregating haplotype refined the critical region to 6.03 Mbp containing 21 protein-coding genes. Whole-genome sequencing uncovered 114 noncoding variants from which CADD predicted one was highly deleterious, a novel substitution within intron-1 of the *sonic hedgehog signaling molecule* (*SHH*) gene. ENCODE data suggested this site was a putative enhancer, subsequently confirmed by luciferase reporter assays with variant-associated gene overexpression.

**Conclusions:**

In a large pedigree, we have identified a *SHH* intron variant that co-segregates with an unusual pediatric cortical cataract phenotype. SHH is important for lens formation, and mutations in its receptor (*PTCH1*) cause syndromic cataract. Our data implicate increased function of an enhancer important for SHH expression primarily within developing eye tissues.

In 2011, we described a large Australian family of European descent that variably presented with four ocular phenotypes: (1) pediatric cortical cataracts (PCCs), (2) familial exudative vitreoretinopathy (FEVR), (3) asymmetric myopia with astigmatism (AM+A), and (4) primary open-angle glaucoma (POAG).^1^ Inspection of the pedigree revealed that the PCC phenotype appeared to segregate in an autosomal dominant inheritance pattern, suggesting a single genetic cause.

Herein, we report the identification of a single disease-linked region and co-segregating haplotype at the telomeric end of chromosome 7. We describe exome and whole-genome sequencing (WGS) approaches that led to the discovery of a predicted highly deleterious novel substitution within intron-1 of the *sonic hedgehog signaling molecule* (*SHH*) gene. We illustrate our application of Encyclopedia of DNA Elements (ENCODE) project data that implicated the intronic region as a candidate *cis*-regulatory element (cCRE) important for *SHH* gene expression selectively in eye tissues during development. Finally, we show confirmation of enhancer function and the effect of the novel intron variant on gene expression using a cell-based reporter assay. Together, these data implicate eye-specific perturbations in *SHH* expression during development as the molecular cause of the unusual PCC phenotype observed in this family.

## Methods

### Human Study Participants

Study subject recruitment occurred as previously described.^1^ Ethical approval was obtained from the Royal Hobart and Royal Victorian Eye and Ear hospitals. All efforts were conducted in accordance with the tenets of the Declaration of Helsinki.

### Exome Sequencing and Coding Variant Filtering

**Figure 1.**
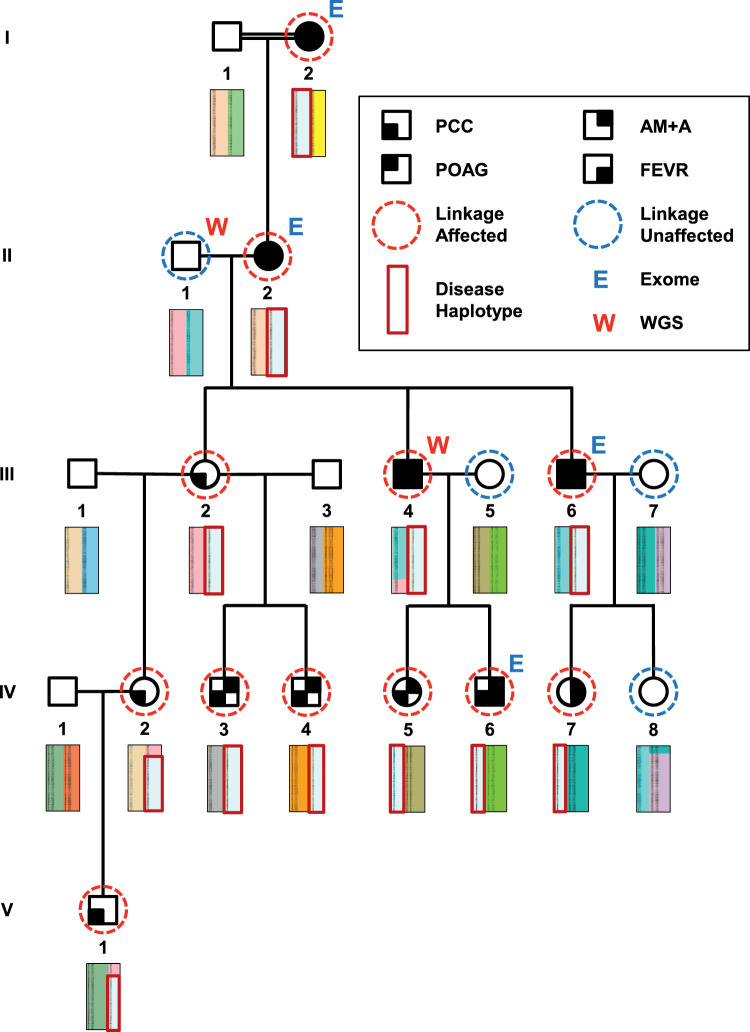
Family pedigree showing clinical phenotypes, linkage analysis affection status, haplotype co-segregation, and sequenced individuals. PCC phenotypes are shown with potential comorbidities (POAG, AM+A, and FEVR), *shaded* according to key. Individuals genotyped for linkage analysis are shown as affected (*red dashed circle*) or unaffected (*blue dashed circle*). Haplotypes were imputed for four unaffected individuals where DNA was unavailable (*no dashed circle*). Haplotype blocks identified within the disease-linked genomic region are depicted as rectangles below each individual with *colors* distinguishing different chromosomal runs of SNP marker alleles. A haplotype block fully co-segregating with disease is highlighted (*dark red box*). In individual IV:2, a recombination event was identified that refined the critical disease interval. Individuals selected for exome (E) and WGS (W) are shown.

Four affected individuals (I-2, II-2, III-6, and IV-6) (Fig. 1) were exome sequenced using a SeqCap EZ Exome V3 capture kit (Roche Nimblegen, Pleasanton, CA, USA) and HiSeq 2500 platform (2 × 100 bp, 100× coverage; Illumina, San Diego, CA, USA). Reads were aligned to the Genome Reference Consortium human genome assembly 37 (GRCh37/hg19; https://www.ncbi.nlm.nih.gov/grc/human) using the Burrows–Wheeler Aligner (BWA 0.5.10; https://sourceforge.net/projects/bio-bwa). PCR duplicates were removed with Picard 1.59 (https://sourceforge.net/projects/picard). Single nucleotide variants (SNVs) and insertion/deletions (indels) were called with the Genome Analysis Toolkit (GATK) 1.6 (https://gatk.broadinstitute.org), and variant filtering/analysis was performed using SNP & Variation Suite (SVS) 8 (Golden Helix, Bozeman, MT, USA; https://www.goldenhelix.com/products/SNP_Variation). An in-house dataset of exome variants identified in 119 individuals with unrelated disease was utilized to filter unrelated variants. Variant allele frequency data were acquired from the Genome Aggregation Database (gnomAD) 2.1.1 release (https://gnomad.broadinstitute.org).^2^ Combined Annotation Dependent Depletion (CADD; https://cadd.gs.washington.edu) was utilized to score the deleteriousness of SNVs and indels, and variants with a score under 20 (not in the top 1% deleterious genome variants) were removed from further analysis.^3^

### Exon Trapping Assay

Exon trapping was performed as previously described.^4^^,^^5^ A 3306-bp genomic fragment containing *GALNT11* exons 11 and 12 was PCR amplified from individual III-5 (forward, 5′-GACTTACGAGCCATAGGAATTTGT-3′; reverse, 5′-TCACACAGCTCAAGCTAAGAAACT-3′). Amplicons with and without the putative exonic splicing enhancer (ESE) variant (c.1788C>T in exon 12, p.C596C) in exon 12 were subcloned into the pSPL3 exon trapping vector (Thermo Fisher Scientific, Waltham, MA, USA) using the XhoI and EcoRV restriction endonuclease sites.

### Genotyping, Multipoint Linkage Analysis, and Haplotyping

Twelve affected and four unaffected individuals were genotyped at 713,014 single nucleotide polymorphisms (SNPs) using Human OmniExpress-24 1.1 BeadChips (Illumina) (Fig. 1). The 103,311 highest quality and informative markers were selected with GenomeStudio Software (Illumina). Genome-wide, multipoint, parametric linkage analysis was performed utilizing Superlink-Online SNP 1.1 (Technion, Haifa, Israel; http://cbl-hap.cs.technion.ac.il/superlink-snp).^6^ A dominant inheritance model was applied with a disease penetrance of 0.99, and linkage was calculated via a hidden Markov model algorithm using a random subset of approximately 1000 markers per chromosome (23,359 SNPs total). The Superlink-Online SNP software was subsequently employed to identify haplotype blocks for markers located within the disease-linked region. Individual haplotypes were assessed for co-segregation with disease throughout the pedigree. The disease-associated haplotype was reviewed for recombination events in all affected individuals to enable refinement of the critical disease interval.

### Whole-Genome Sequencing and Noncoding Variant Filtering

WGS was performed on an affected individual and his unaffected father (II-1 and III-4, respectively) (Fig. 1) using an Illumina HiSeq X Ten platform (2 × 150 bp, 30× coverage; Novogene Corporation, Sacramento, CA, USA). Reads were aligned to reference genome GRCh37/hg19 with the BWA, SNVs and indels were detected with GATK, structural variants (SVs) were detected with Delly (https://github.com/dellytools/delly), and copy number variants (CNVs) were detected with the Control-FREE Copy number and allelic content caller (Control-FREEC; https://github.com/BoevaLab/FREEC). Rare variants were identified using the Golden Helix SVS software. CADD analysis was used to prioritize variants by their predicted deleteriousness.

### Assessment of Enhancer Function

Putative enhancer function and the effects of the *SHH* intron variant were determined using the Dual-Glo Luciferase Assay system (Promega, Madison, WI, USA) according to the manufacturer's protocol. A 296-bp genomic region including the 221-bp putative intronic regulatory element (chr7:155808575-155808796, GRCh38; chr7:155601269-155601490, GRCh37) was PCR amplified from affected individual III-4 using primers incorporating NheI and HindIII restriction endonuclease sites for subsequent cloning (forward, 5′-CTGACTGAGCTAGCGAGGCCGAGGGTTGCTGGAGTTGG-3′; reverse, 5′-TCAGTCAGAAGCTTCGGCTCGCAGATCAGGGAGGTAGG-3′). The genomic fragments were ligated into vector pGL4.24 (Promega), upstream of a firefly luciferase reporter gene containing a minimal promoter. Three experimental conditions were tested using constructs including reference, variant, or no putative regulatory element sequence (empty vector). Experimental constructs were transfected into human hepatocellular carcinoma cells (HepG2; American Type Culture Collection, Manassas, VA, USA) in parallel with a *Renilla* luciferase control plasmid (pGL4.73 [hRluc/SV40]; Promega) for subsequent normalization of transfection efficiencies. Twenty-four biological replicates were performed for each condition. Average response ratios were calculated from experimental versus control luminescence signals relative to the reference condition. Standard errors were calculated, and statistical significance was determined using the paired *t*-test.

## Results

### Core Pedigree Identified With an Unusual Dominantly Inherited PCC

**Figure 2.**
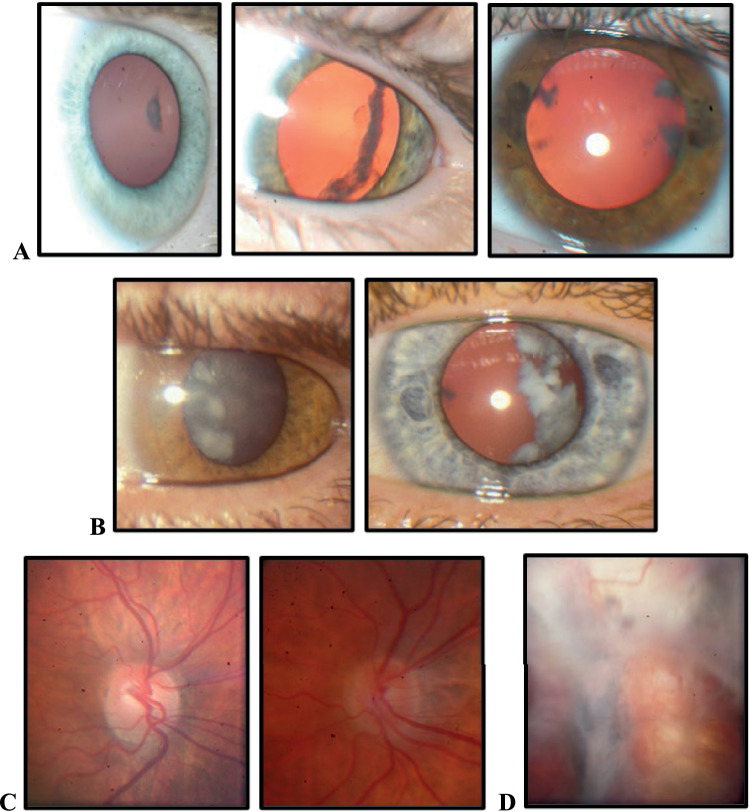
PCC phenotype observed in all affected family members and FEVR noted in a subset of affected individuals. (**A**) PCC is mild in individuals between the ages of 5 and 9. (**B**) PCC is more extensive at 38 and 40 years of age (*middle row*). (**C**) Dragged retinal vessels and (**D**) severe retinal detachment postsurgery at 16 years of age in individuals with FEVR.

We previously reported a detailed description of the family's clinical phenotypes in a 63-person extended pedigree.^1^ Herein, we investigated the molecular basis of disease in a 20-person core portion of the pedigree (Fig. 1, Table 1) primarily affected with PCCs, which were observed in 11 individuals (Figs. 2A, 2B). For individual IV-7, we could not describe her cataract features, as she underwent bilateral lensectomies at a young age prior to ascertainment. We classified her as affected due to having FEVR and AM+A clinical features shared with five affected relatives with PCCs. Six individuals were affected with FEVR, of whom five had dragged retinal vessels (Fig. 2C), and another developed a severe retinal detachment (Fig. 2D). Four individuals had POAG, and eight individuals had AM+A of at least –3 diopters (D) in one eye. The pedigree showed autosomal dominant transmission of PCC through all five generations. Segregation of the three additional ocular phenotypes (FEVR, AM+A, and POAG) was less consistent, but we included them as potential comorbidities.

**Table 1.** Clinical Features of Affected Family Members Examined, Ordered by Ascending Current Age

					Potential Comorbidities			
Core Pedigree ID	Original Pedigree ID^1^	Current Age (y)	Age at First Exam (y)	PCC	POAG	AM+A	Notes	FEVR
IV-5	New	13	1	Yes	No	Yes	No	—
V-1	IX:1	15	3	Yes	No	No	No	—
IV-6 E	VIII:7	26	8	Yes	No	Yes	DD	IA
IV-4	VIII:6	28	12	Yes	No	Yes	No	—
IV-3	VIII:5	32	16	Yes	No	Yes	No	—
IV-7	VIII:8	33	17	I	I	I	TRD (OU)	CL (OU)
IV-1	VIII:3	37	25	Yes	No	No	No	—
III-6 E	VII:7	52	36	Yes	Yes	Yes	DD	CS
III-4 W	VII:5	55	39	Yes	Yes	Yes	DD	CS
III-2	VII:3	59	43	Yes	No	No	No	—
II-2 E	VI:7	81	65	Yes	Yes	Yes	DD	—
I-2 E	V:4	Deceased	83	Yes	Yes	Yes	DD	—

PCC, pediatric cortical cataract; DD, dragged disc; IA, iris atrophy; I, indeterminable; OU, both eyes; TRD, total retinal detachment; CL, childhood lensectomy; CS, cataract surgery.

### Exclusion of Known Disease Genes

To identify candidate disease-causing variants in the coding regions of the genome, we performed exome sequencing on affected individuals spanning four generations (Fig. 1). As the reported prevalence of congenital/childhood cataract ranges from 0.95 to 32.64 per 10,000 children, we excluded all variants with an allele frequency greater than 1 in 10,000 in any specific population within the gnomAD database.^7^ The coding regions of all 41 known congenital cataract genes (*AGK*, *BFSP1*, *BFSP2*, *CHMP4B*, *CRYAA*, *CRYAB*, *CRYBA1*, *CRYBA4*, *CRYBB1*, *CRYBB2*, *CRYBB3*, *CRYGB*, *CRYGC*, *CRYGD*, *CRYGS*, *CTDP1*, *ELP4*, *EPHA2*, *EYA1*, *FAM126A*, *FOXE3*, *FYCO1*, *GALK1*, *GCNT2*, *GJA3*, *GJA8*, *HSF4*, *LIM2*, *MAF*, *MIP*, *MIR184*, *NHS*, *P3H2*, *PAX6*, *PITX3*, *PXDN*, *SIL1*, *SLC16A12*, *SLC33A1*, *TDRD7*, and *VIM*) were well covered in all four exome datasets, but no shared rare variants were identified. Rare variants within the seven known genes/loci associated with exudative vitreoretinopathy (Online Mendelian Inheritance in Man [OMIM], EVR1–EVR7) were also excluded. The X-linked FEVR gene *NDP* was excluded due to male-to-male disease transmission observed in the pedigree. Variants were also excluded from the five other known FEVR genes (*FZD4*, *LRP5*, *TSPAN12*, *ZNF408*, and *CTNNB1*). Finally, shared rare coding variants were not identified within the exudative vitreoretinopathy 3 locus (OMIM# 605750; chr11:31000000-43400000, GRCh38) in which the disease gene has yet to be determined. Rare variants within the myocilin (*MYOC*) gene (OMIM# 601652), which is responsible for the majority of Mendelian POAG, were also excluded.

### Single Candidate Coding Variant in *GALNT11* Excluded

**Figure 3.**
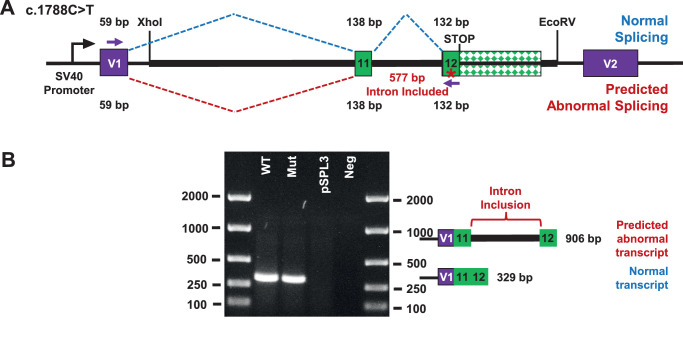
Exon trapping assay. (**A**) Wild-type (WT) and variant (Mut) genomic DNA fragments were cloned between pSPL3 expression vector exons V1 and V2. Mini-gene constructs were transfected into Cos-7 cells, mRNA was collected after 24 hours, and cDNA was synthesized. (**B**) PCR amplification using primers within vector exon V1 and insert exon 12 showed that both WT and Mut transcripts were normally spliced. pSPL3, cDNA from cells transfected with empty vector; Neg, cDNA from non-transfected cells.

Overall, exome sequencing identified only one rare coding variant shared by all four affected individuals—a c.1788C>T (NM_022087.4) change in the *GALNT11* gene that was synonymous (p.C596C). The variant fully co-segregated with the affection status of all core family members. The variant is known (dbSNP rs374094445) but observed at low frequency, with the highest observed in Africans and African Americans (minor allele frequency [MAF], 0.00008). *GALNT11* showed the strongest tissue expression in kidney, pancreas, and retina (Genotype-Tissue Expression project), consistent with a role in the eye. Analysis of the synonymous change using ESE Finder predicted destruction of an ESE (serine/arginine rich splicing factor 1 binding site) within exon 12. However, an exon-trapping assay that utilized expression of reference and variant *GALNT11* mini-gene constructs in Cos-7 cells determined that the resulting transcript species were spliced identically (Fig. 3) and indicated that the *GALNT11* variant was functionally benign.

### Disease Locus Identified on Chromosome 7q36

**Figure 4.**
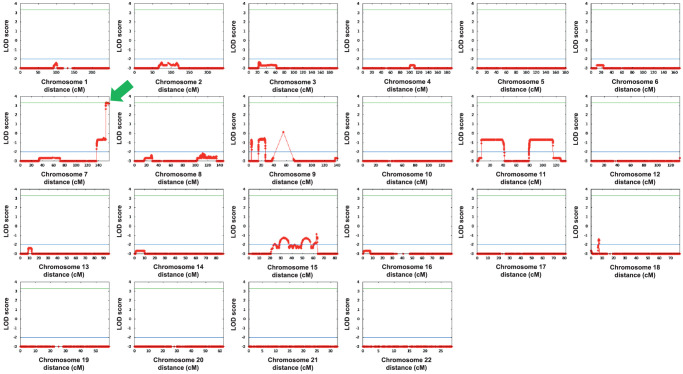
Identification of the disease-associated locus. Multipoint linkage analysis using a selection of 23,359 high-quality and informative SNPs identified a single linked region at the telomeric end of chromosome 7 (7q36, *green arrow*) with a maximum LOD score of 3.3.

To locate the chromosomal location of the disease-causing gene, we previously performed linkage analyses on the larger family pedigree using 6008 genome-wide SNPs (Linkage Panel IVb, Illumina).^1^ Two-point analysis using FastLink software^8^^,^^9^ and multipoint analysis using MERLIN software^10^ revealed multiple SNPs of interest but no statistically significant focal localization. In this study, we employed high-density BeadChips to genotype 713,014 SNPs in individuals throughout the core pedigree. Following selection of the 103,311 highest quality and informative markers, multipoint linkage analysis was performed on a random subset of 23,359 genome-wide SNPs using Superlink-Online SNP software. This approach identified a single 6.23-centimorgan (cM) support interval located at the telomeric end of chromosome 7q36 with a maximum logarithm of the odds (LOD) score of 3.3 (LODmax, rs6966462; 1 LOD unit support interval, rs1525041 to telomere) (Fig. 4). Haplotype analysis of SNP markers across the linked region identified a single haplotype block co-segregating with disease in all 12 affected individuals and none of the unaffected family members (Fig. 1). A recombination event between markers rs396672 and rs6960275 discovered in individual IV:1 and their son (V:1) refined the centromeric end of the disease interval (chr7:153017006-159138663, GRCh37; chr7:153319921-159345973, GRCh38) to a 6.03-Mbp region containing 21 protein-coding genes.

### Novel Noncoding Variant Identified in Intron-1 of *SHH*

As exome sequencing had not identified the pathogenic variant within the coding regions of the genome, we performed WGS to discover additional variants located within uncaptured coding exons and gene regulatory regions such as promoters and enhancers. Two individuals were chosen for WGS based on their known disease haplotype status—a father lacking the disease haplotype and his son who carried the disease haplotype (II-1 and III-4, respectively) (Fig. 1). Sequencing these two individuals identified all variants located within the disease haplotype by excluding those detected in the unaffected father from those discovered in the affected son. Variants observed in three unrelated WGS samples were also excluded.

**Figure 5.**
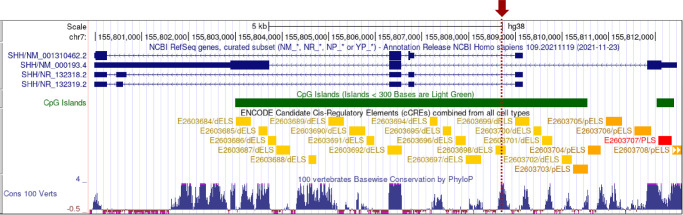
Rare deleterious noncoding variant (*red arrow*/*dotted line*) is located within intron-1 of the *SHH* gene. Data tracks from the UCSC Genome Browser are shown. The *SHH* gene expresses two protein-coding and two noncoding transcripts from alternative promoters (*blue bars*). The intronic variant (chr7:155808703G>A, GRCh38; chr7:155601397G>A, GRCh37) is 290 bp from the first exon of transcript-2 (NM_001310462.2), located within a CpG island (*green bar*), where ENCODE data predict a regulatory element with a distal enhancer-like signature (dELS, E2603698; *yellow bar*) that is highly conserved in vertebrates (*blue Manhattan plot peak*).

Interestingly, WGS did not identify any rare coding variants located within the disease interval, a tribute to exome-capture methods. As expected, WGS detected many noncoding variants (Table 2). Remarkably, out of the 44 rare noncoding SNVs and indels identified, the CADD algorithm determined only one to be within the top 1% of deleterious variants in the genome (CADD score 21.7), a SNV located within intron-1 of the *SHH* gene (Fig. 5). The *SHH* gene expresses two protein-coding and two noncoding transcripts from alternative promoters. The intronic variant was located closest (290 bp) to the first exon of coding transcript-2. The potential pathogenicity of the variant was supported by its novelty, not being observed in more than 152,000 alleles in the gnomAD v3.1.2 database (WGS data). The intron variant was located within a highly conserved region, with the reference nucleotide shared by 74 of 81 vertebrate species with sequence data at this location, suggesting functional significance. The importance of this noncoding region for gene regulation was further implicated by its location within a CpG island and a regulatory element predicted by the ENCODE project.^11^^,^^12^

**Table 2.** Summary of Variants Identified Through WGS

Variant Type	Number Identified
Rare coding variants (SNVs, indels, SVs, or CNVs)	0
Noncoding SVs	55 (53 intronic, 2 intergenic)
Noncoding CNVs	15 (9 intronic, 6 intergenic)
Rare noncoding SNVs	38 (24 intronic, 14 intergenic)
Rare noncoding indels	6 (6 intronic; 3 del, 3 ins)
Rare noncoding SNVs and indels with CADD score > 20	1

We defined rare as MAF < 0.0001.

**Figure 6.**
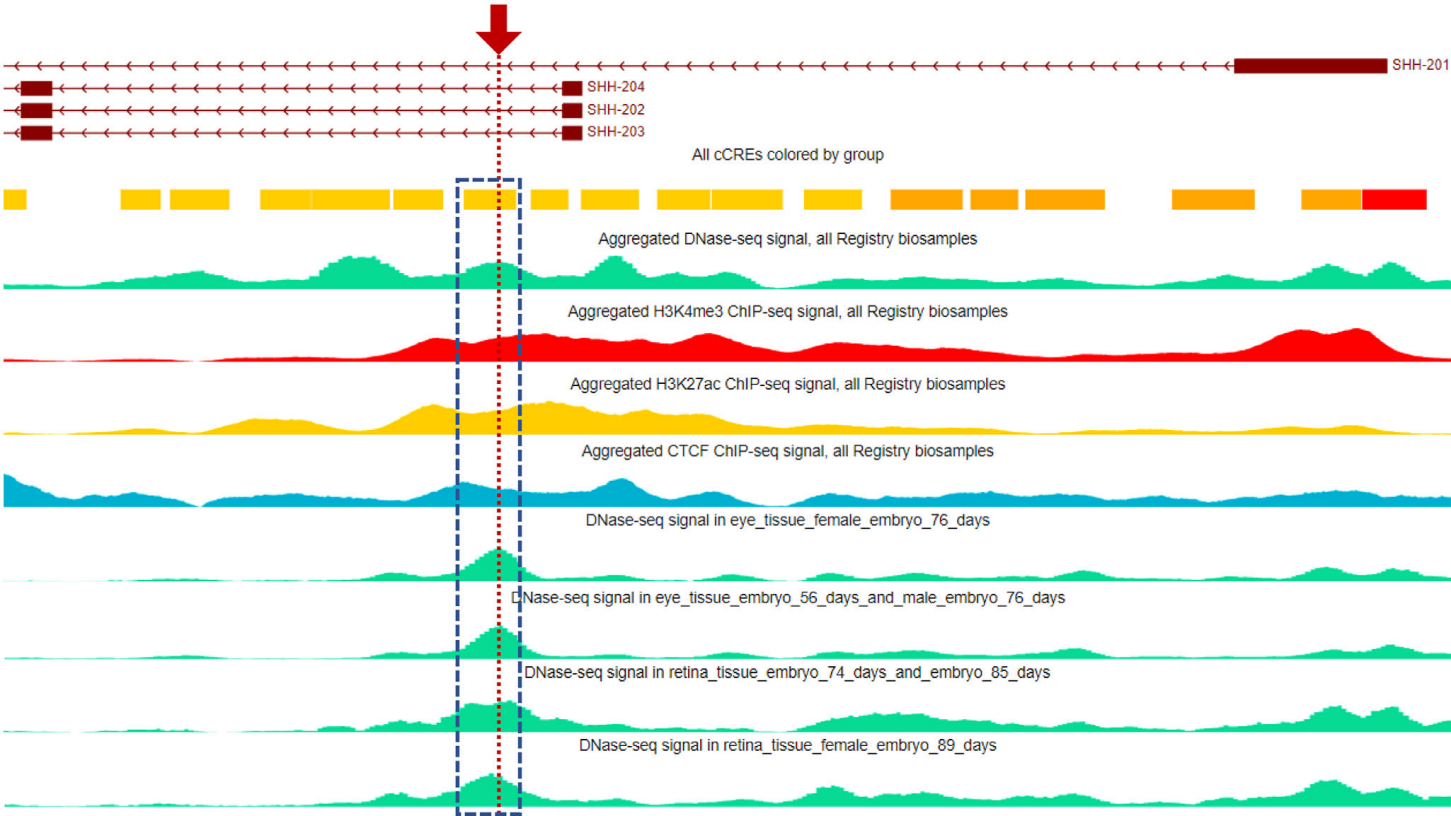
ENCODE data predict the variant is located within a distal cCRE important for eye development. Combined cell data of signals for DNase hypersensitivity (*green*), H3K4me3 (*red*) and H3K27ac (*yellow*) histone modifications, and CTCF binding (*blue*) implicate a 221-bp cCRE (*yellow bar* with associated data boxed in *blue*) distal to *SHH* transcriptional start sites. Highest DNase hypersensitivity signals were derived from embryonic eye and retina tissues (*lower four green plots*). The location of the novel deleterious intronic variant is denoted (*red arrow*/*dotted line*).

Inspection of ENCODE data within the SCREEN Registry (version 3; https://screen.wenglab.org/) showed the intronic variant was located within a 221-bp candidate cCRE with a distal enhancer-like signature (dELS; EH38E2603698) (Fig. 6). The ENCODE project identifies and classifies potential regulatory elements according to four biochemical signatures obtained from human cell lines and tissues. Primarily the region must be in an open chromatin state, making the site DNase hypersensitive, which can be determined by DNase-seq experiments. Secondary support is provided by chromatin immunoprecipitation sequencing (ChIP-Seq) experiments that identify histone modifications (H3K4me3 and H3K27ac) and CCCTC-binding factor (CTCF) binding that are important for regulating chromatin state and gene expression. The intron variant was within a cCRE with high signals for all four biochemical signatures when data were combined from all ENCODE biosamples (maximum *z*-scores: DNase, 3.31; H3K4me3, 4.59; H3K27ac, 4.28; CTCF, 2.98). Furthermore, out of 1118 human tissues profiled, the top two highest DNase-seq signals detected at the cCRE element were derived from tissues of 76-day female embryonic eye (*z*-score, 3.31) and 56-day male embryonic eye (*z*-score, 3.18). Retina tissue from a 74-day embryo provided the fifth highest DNase-seq signal (*z*-score, 3.14), and an 89-day female embryonic retina tissue was 5th (*z*-score, 2.96). Taken together, these data provide strong evidence to support the specific importance of the cCRE during eye development.

### Novel Intronic Variant Effects a *SHH* Distal Enhancer

To test whether the cCRE could influence gene expression and to determine the effect of the novel intronic variant, we performed luciferase reporter assays, which placed the reference and variant element sequences upstream of a firefly luciferase gene with a minimal promoter. For an enhancer to function effectively, transcription factors (TFs) are required that bind to the enhancer sequence and aid in the recruitment of RNA polymerase II to the promoter. Experimental data from the ENCODE project showed that 19 different TFs, including CTCF, bind to the cCRE sequence, but artificially creating such molecular environmental conditions in cultured cells would have proven problematic. Further interrogation of the ENCODE data showed that, out of 28 cell lines profiled, the highest biochemical signals observed for all four enhancer signatures were identified in a commercially available human liver carcinoma cell line, HepG2 (*z*-scores: DNase, 3.19; H3K4me3, 4.05; H3K27ac, 3.08; CTCF, –10.00). Consequently, we employed HepG2 cells to endogenously provide the TFs critical for enhancer function in our luciferase reporter experiments.

**Figure 7.**
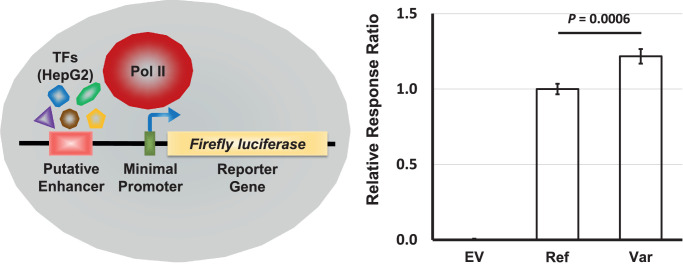
Assessment of putative enhancer function and variant effects. (*Left*) Reference and variant enhancer sequences were engineered upstream of a firefly luciferase reporter gene associated with a minimal promoter. Constructs were transfected into HepG2 cells that endogenously provided TFs to aid in the recruitment of RNA polymerase II to the promoter. (*Right*) The presence of either the reference or variant element greatly increased the levels of reporter expression, confirming enhancer function. Compared to the reference enhancer, the variant form led to significant overexpression of the reporter gene. Results from each condition are an average of 24 biological replicates. *Standard error bars* are provided with statistical significance determined by paired *t*-test.

The luciferase assay results (Fig. 7) showed that the presence of either the reference or variant cCRE sequence greatly increased the levels of reporter expression, confirming the intronic element as a legitimate enhancer. Interestingly, compared to the reference enhancer, the variant form caused significant overexpression of the reporter gene. Together, these data support altered *SHH* gene expression as a functional consequence of the intronic variant.

## Discussion

SHH is the most widely studied ligand of the hedgehog signaling pathway and a well-known classical morphogen, inducing embryonic cell targets to differentiate into specific cellular identities according to the precise levels and duration of its signaling.^13^ The mechanism of SHH signaling can be described as “double-negative” activation.^14^ In the presence of SHH ligand, its binding to the Patched (PTCH) transmembrane receptor relieves repression on a second transmembrane protein, Smoothened (SMO).^13^ Activated SMO then initiates intracellular signal transduction resulting in GLI family transcription factor processing into their active forms (GLIA) and subsequent transcription of SHH target genes. In the absence of SHH signal, the PTCH receptor inhibits SMO, and the GLI proteins are either degraded or processed into transcriptional repressors (GLIRs). GLI-targeted genes include *PTCH1* itself, thus creating a negative feedback loop that represses SHH pathway activation through limiting ligand dispersion and signal transducer activity.^15^ SHH can signal in an autocrine fashion, affecting the cells in which it is produced, or it can act as a paracrine signal to induce changes in other cells. Further regulation of paracrine signaling is provided by the Dispatched (DISP) protein, which facilitates the extracellular secretion of SHH.^16^ The action of SHH in defined concentration gradients and spatiotemporal patterns is crucial for organizing many developing tissues, including the eye, craniofacial structures, brain, spinal cord, and limbs.^17^ In addition, SHH signaling regulates adult stem cells involved in the maintenance and regeneration of adult tissues.^18^

In humans, mutations in *SHH* have been reported to cause a wide range of developmental abnormalities. The severe end of the disease spectrum includes massive brain and ocular malformations, such as holoprosencephaly (OMIM 236100), where the forebrain fails to separate into two hemispheres, accompanied by cyclopia, anophthalmia, or microphthalmia.^19^^,^^20^ At the mild end of the disease spectrum, *SHH* mutations have resulted in more subtle effects on specific eye components, such as isolated iris and uveal-retinal coloboma, with or without microphthalmia.^21^^–^^23^

SHH signaling plays a crucial role in eye vesicle patterning in vertebrates. SHH promotes expression of PAX2 in the optic stalk and represses expression of PAX6 in the optic cup.^24^ SHH signaling contributes to establishment of both proximal–distal and dorsal–ventral axes by activating VAX1, VAX2, and PAX2.^25^ In the dorsal part of the developing retina, BMP4 is expressed and antagonizes the ventralizing effects of SHH signaling.^26^ In human embryos, *SHH* expression has been demonstrated in the developing optic vesicle, superficial lens, and posterior retina.^27^ SHH is also important for the differentiation of periocular neural crest (PNC) cells, which are indispensable for anterior chamber development.^20^ PNCs interact with the forming cornea and lens and define the edge of the optic cup from which the ciliary body, Schlemm's canal, and iris are formed.^28^

Studies support an association between abnormal SHH signaling and the PCC phenotype observed in the family described here. Although there do not appear to be reports of nonsyndromic cataracts caused by *SHH* mutations in the literature, the SHH pathway is important for lens development. The SHH ligand, PTCH receptor, and BMP4 antagonist are all expressed in the developing lens.^27^ Congenital cataracts are found in 18% of patients with Gorlin–Goltz syndrome, which is caused by mutations in the SHH receptor gene *PTCH1*.^29^ BMP4 is expressed strongly in the optic vesicle and weakly in the surrounding mesenchyme and surface ectoderm, where it has crucial roles during lens induction.^30^ SHH signaling plays a key role in cataract development and in the response of normal lenses to radiation injury. Mice heterozygous for *Ptch1* develop spontaneous cataracts and are highly susceptible to cataract induction by exposure to ionizing radiation at an early postnatal age, when lens epithelial cells undergo rapid expansion in the lens epithelium.^31^

FEVR is characterized by variable degrees of avascular peripheral retina, extraretinal neovascularization, macular dragging, and retinal detachment. This phenotype was observed in six of the 12 (50%) individuals carrying the *SHH* variant and was not detected in four family members without the variant. We are unable to provide further genetic evidence to support a causative relationship between the *SHH* variant and this phenotype but include it for the purpose of providing information regarding FEVR as a possible comorbidity. It should be noted that the SHH pathway is an important component of normal retinal angiogenesis with activation of *Shh* leading to an increase in angiogenic factors (angiopoietin-1 and angiopoietin-2),^32^ and SHH blockade inhibiting vascular neogenesis.^33^ Mutations in the *PTCH1* gene can lead to an abnormal intraretinal glial response that stimulates the retinal surface to proliferate and contract, creating retinal membranes, dragged vessels, and macular holes in patients with Gorlin–Goltz syndrome.^29^^,^^34^^–^^37^ Similar abnormalities are exhibited in mice lacking a *Ptch1* allele, wherein abnormal vitreoretinal cell cycle regulation leads to photoreceptor dysplasia and Müller cell-derived gliosis.^38^ Funnel retinal detachment has also been reported in patients with *BMP4* mutations.^27^

The AM+A phenotype was observed in 73% (8/11) of assessed *SHH* variant carriers and is likely a secondary result of the cataracts distorting the focus of the lens. Due to the childhood onset of the cataracts, they may also affect visual cues involved with retinoscleral signaling during emmetropization, leading to abnormal scleral remodeling of ocular shape and further refractive error. The environment is a well-established modifier of refractive error status, which may also explain the reduced penetrance of this potential comorbidity. It is worth noting that myopia appears to be common in patients with holoprosencephaly, with one study observing five of 10 patients (50%) with moderate myopia.^39^ Myopia is also commonly observed in Gorlin–Goltz syndrome, with one report describing nine of 11 patients (82%) affected by various degrees of myopia, ranging from −0.5 to −10 D.^29^ Two patients showed high anisometropia (18%), with a 6-D difference in one patient and 10-D difference in the other; only two patients (18%) were emmetropes. In guinea pigs, the SHH signaling pathway has been shown to induce myopia by activating matrix metalloproteinase 2.^40^ In chicks with experimentally induced myopia, SHH expression is increased in the retina, which suggests its involvement in the retinoscleral feedback that controls postnatal eye growth.^41^

POAG affects pedigree members over 40 years of age, so we cannot ascribe affection status to younger family members. This limits the genetic evidence to support an association between POAG and the *SHH* variant identified in this family. Currently, four of the five (80%) *SHH* variant carriers over the age of 40 have developed POAG, and we expect that more younger carriers will develop glaucoma as they age (Table 1). *SHH* has not previously been associated with glaucoma, but the involvement of the pathway in the differentiation of PNC cells, which are important for anterior chamber development and subsequent ciliary body, Schlemm's canal, and iris formation, make it an obvious candidate.^20^^,^^28^ Subtle developmental defects in these structures could lead to late-onset failure of aqueous outflow resulting in POAG. SHH also plays a role in early retinal ganglion cell (RGC) development, as it is secreted by differentiated RGCs to induce its own expression and promote the differentiation of retinal precursor cells into further RGCs.^42^^–^^44^ Additionally, the precise regulation of SHH levels inside the retina is critical, as it stimulates RGC axon growth at low concentrations but inhibits growth at high concentrations.^45^

The SHH enhancer affected in this family appears to be important primarily during eye tissue development, which explains the lack of non-ocular phenotypes normally associated with mutations in the *SHH* gene or its molecular partners. There are reported examples of other genes associated with both major ocular developmental abnormalities and non-syndromic childhood or adult cataract. Mutations in the *SOX2* gene are known to account for 20% of anophthalmia and microphthalmia in humans, but markers in proximity to the *SOX2* gene and within its regulator, *SOX2-OT*, have recently been associated with cataract.^46^^–^^48^ Likewise, *PAX6* is well known as a master regulator of eye formation in which mutations have largely been associated with aniridia, but mutations in this gene have also been identified in families with congenital cataracts.^49^^,^^50^

Many long- and short-range enhancers are likely to be selectively used to control the exact concentration and spatiotemporal patterns of *SHH* expression. Importantly, data from the ENCODE project has implicated the distal enhancer presented in this study as being selectively important for human embryonic eye development. Furthermore, our cell-based assay data have shown that the novel intron variant identified in this family causes enhanced gene expression. As *SHH* is known to be important for the development of the lens in humans, for which precise gene expression is critical, we propose that the novel intronic variant is responsible for the unusual PCC phenotype observed in this unique family. FEVR, AM+A, and POAG were not observed in all individuals carrying the *SHH* variant, which may be due to incomplete penetrance. However, further genetic evidence will be required to confirm they can also be caused by *SHH* mis-expression in the eye.

In conclusion, this family report and genetic study expands the ocular phenotypic expression of mutations associated with the *SHH* gene to include PCC. Based on our findings, we recommend investigation of genes within the SHH pathway in families with cataract. The SHH pathway should also be considered in families with glaucoma, myopia, and retinal vasculopathy phenotypes.
